# Supplementary figures and images for: Synergistic Photothermal Therapy and Chemotherapy Enabled by Tumor Microenvironment-Responsive Targeted SWCNT Delivery
Source: Int J Mol Sci. 2024 Aug 23;25(17):9177. doi: 10.3390/ijms25179177 (PMC11394823; doi:10.3390/ijms25179177)

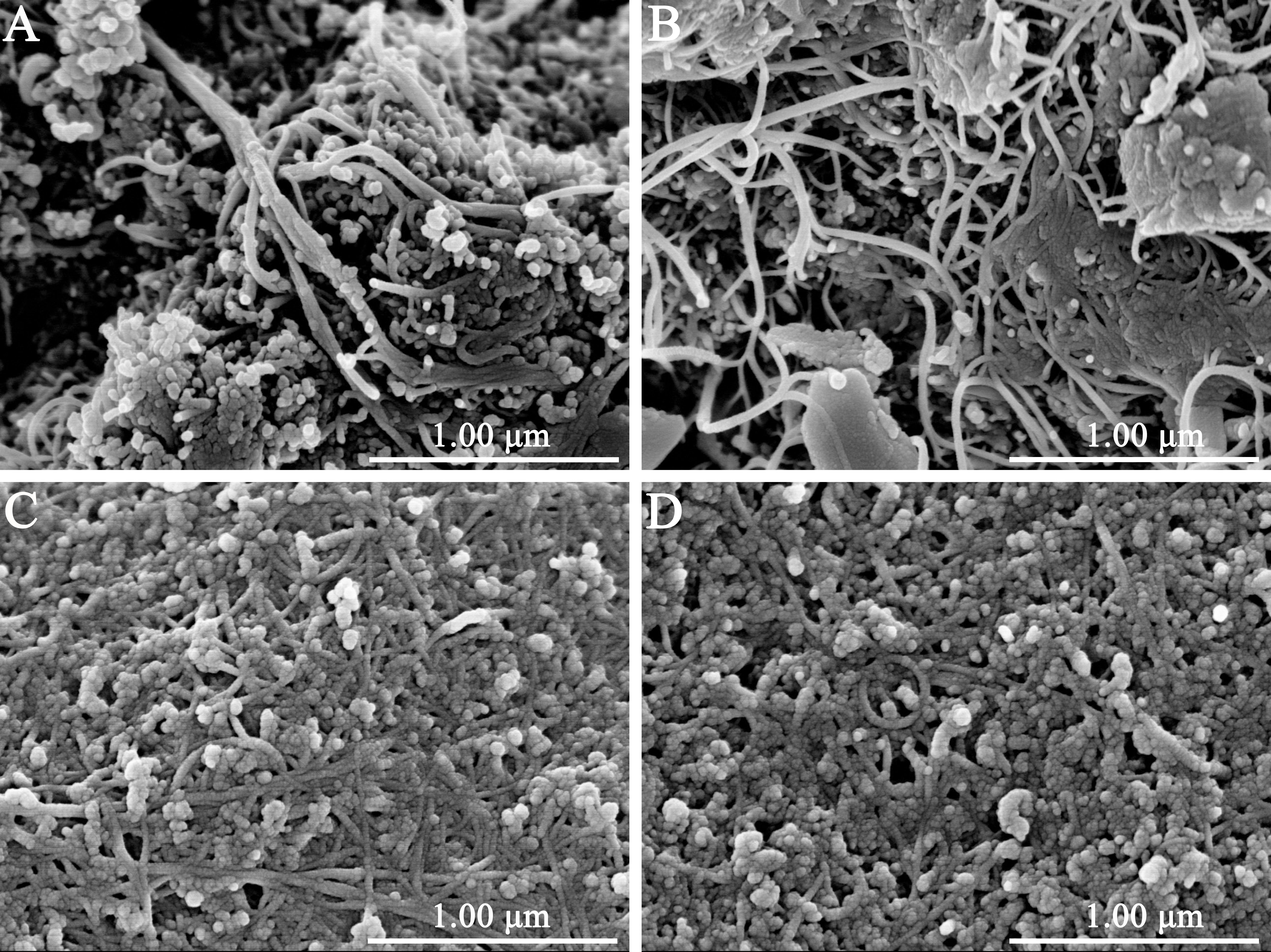

Supplement: Supplementary file 1 [file ijms-25-09177-s001.zip › Supplementary Figures/Fig. S2.jpg]

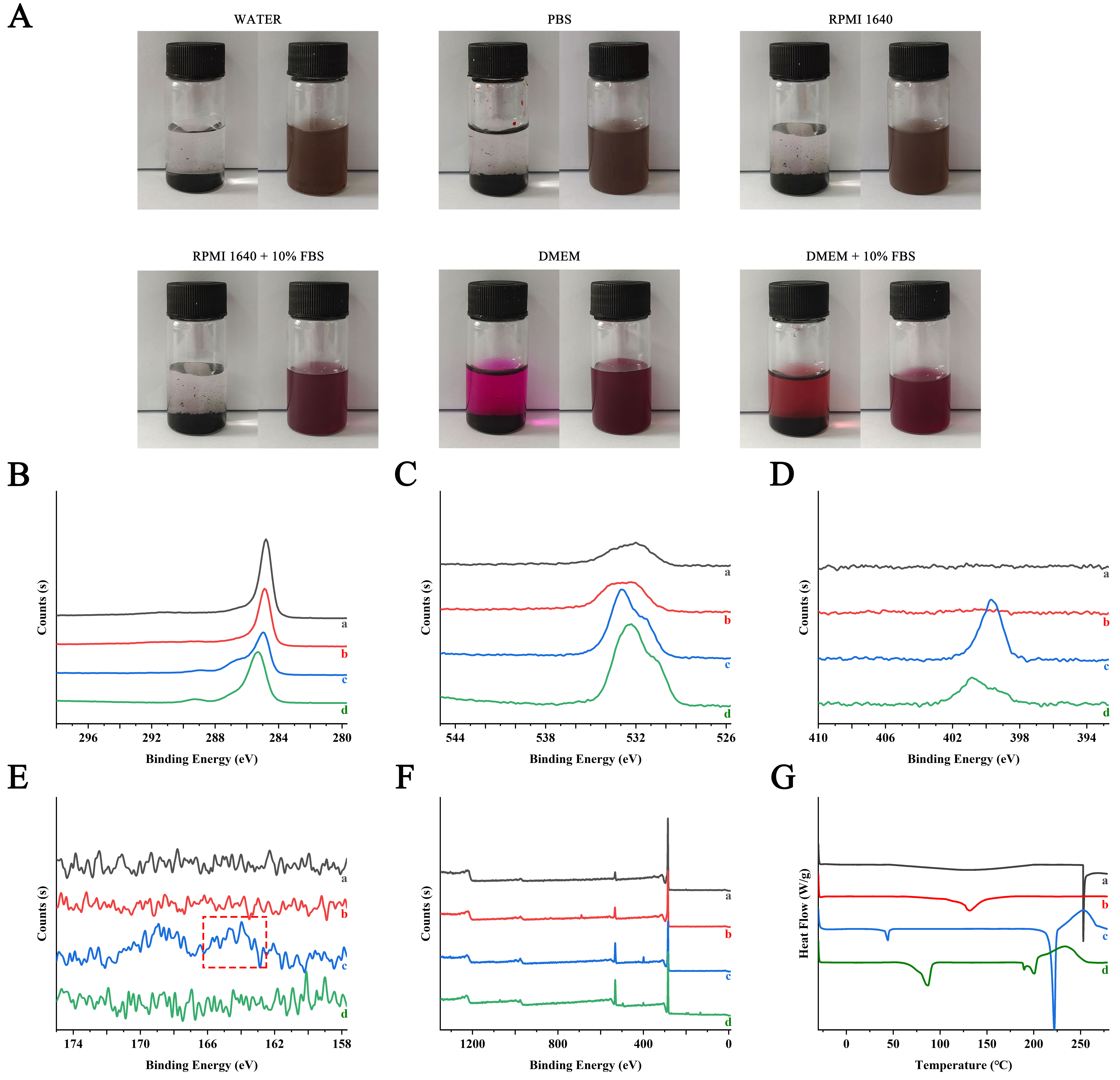

Supplement: Supplementary file 1 [file ijms-25-09177-s001.zip › Supplementary Figures/Fig. S3.jpg]

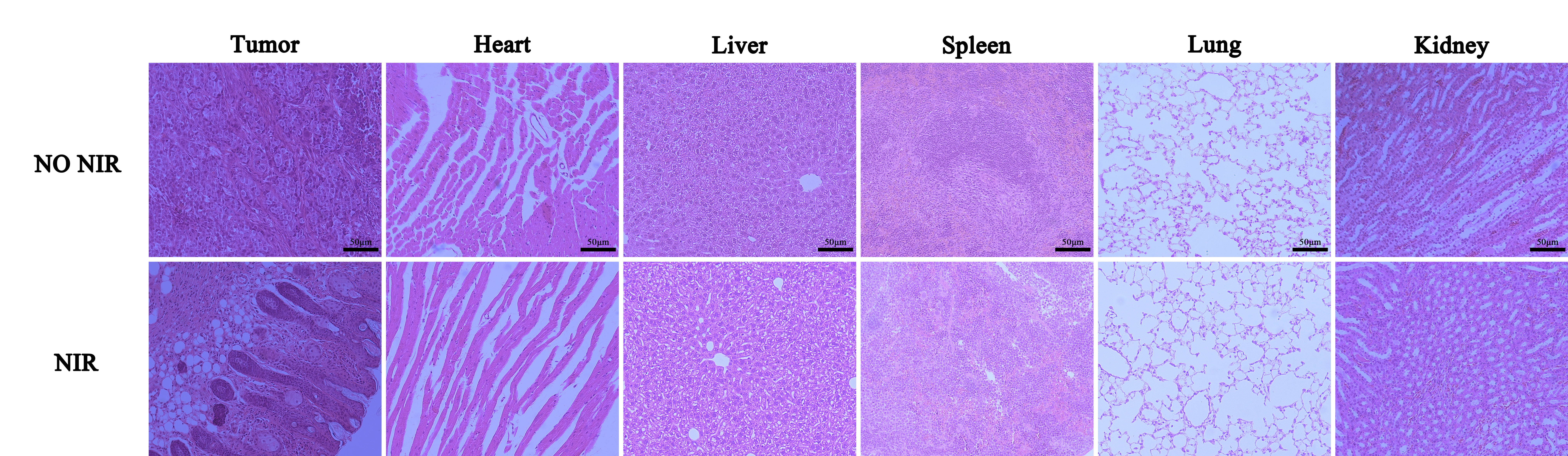

Supplement: Supplementary file 1 [file ijms-25-09177-s001.zip › Supplementary Figures/Fig. S5.jpg]
